# Gene Expression Profiling of *EGFR*, *FGFR2*, *PIK3CA*, *PTEN*, *SMAD4*, *STK11*, and *TP53* in Cell‐Free RNA From Exhaled Breath Condensate: Diagnostic, Prognostic, and Therapeutic Implications in Advanced Lung Adenocarcinoma

**DOI:** 10.1002/cnr2.70566

**Published:** 2026-06-03

**Authors:** Sabina Latifova, Uygar Genis, Haydar Soydaner Karakus, Korcan Korba, Su Ozgur, Ceyda Aldag, Tuncay Goksel, Levent Pelit, Cumhur Gunduz, Asli Tetik Vardarli

**Affiliations:** ^1^ Department of Basic Oncology, Faculty of Medicine Ege University Izmir Türkiye; ^2^ Department of Medical Biology, Faculty of Medicine Ege University Izmir Türkiye; ^3^ Department of Pulmonary Diseases, Faculty of Medicine Ege University Izmir Türkiye; ^4^ Translational Pulmonary Research Center (EgeSAM) Ege University Izmir Türkiye; ^5^ Department of Chemical Technologies, Faculty of Engineering Ege University Izmir Türkiye; ^6^ WHO/IARC‐GICR, Regional Cancer Registry in North Africa, Central and Western Asia Izmir Türkiye; ^7^ Department of Chemistry, Faculty of Science Ege Universit Izmir Türkiye

**Keywords:** adenocarcinoma, cell‐free RNA, EBC, *EGFR*, *FGFR2*, gene expression, *PIK3CA*, *PTEN*, *STK11*, *TP53*

## Abstract

**Background:**

Advanced non‐small cell lung cancer (NSCLC), particularly lung adenocarcinoma, remains a leading cause of cancer‐related mortality. Although molecular profiling has improved disease stratification, most biomarker assays rely on invasive tissue sampling. Exhaled breath condensate (EBC) is an attractive non‐invasive biofluid that may provide clinically relevant molecular information. This study evaluated whether cell‐free RNA (cfRNA) obtained from EBC can capture diagnostically and prognostically informative gene‐expression changes in advanced lung adenocarcinoma.

**Methods:**

In this prospective study, 40 patients with histologically confirmed stage IIIB–IV lung adenocarcinoma and 20 healthy controls were enrolled. EBC samples were collected from all participants, and plasma samples were obtained from a subgroup of 10 patients and 10 controls. Expression of *EGFR, FGFR2, PIK3CA, PTEN, SMAD4, STK11,* and *TP53* was quantified by RT‐qPCR and normalized to GAPDH using the 2^−ΔΔCT^ method. Receiver operating characteristic (ROC) analysis, correlation testing, and Kaplan–Meier survival analysis were performed.

**Results:**

The most clinically relevant findings were observed for *PIK3CA*, *FGFR2*, and *EGFR*. In EBC, *PIK3CA* emerged as the only gene with significant diagnostic performance for distinguishing patients from healthy controls (AUC = 0.8522; sensitivity 81.58%; specificity 76.92%). In survival analyses, high *FGFR2* and high *PIK3CA* expression were associated with significantly shorter overall survival, supporting their prognostic relevance. In treatment‐stratified analysis, low *EGFR* expression was associated with longer survival in patients receiving chemotherapy alone, suggesting potential predictive value in this subgroup. In plasma, *PIK3CA* was upregulated approximately five‐fold, whereas *EGFR* was downregulated approximately five‐fold. Notably, *EGFR* expression showed a strong positive correlation between EBC and plasma (*R* ^2^ = 0.7849, *p* = 0.0006), indicating cross‐matrix consistency for this marker. By contrast, alterations in *PTEN*, *TP53*, and *SMAD4* should be considered exploratory, as they showed differential expression but lacked comparable diagnostic, prognostic, or treatment‐associated strength.

**Conclusions:**

EBC‐based cfRNA profiling provides a feasible, non‐invasive approach for molecular characterization of advanced lung adenocarcinoma. Among the genes examined, *PIK3CA* showed the strongest diagnostic signal, *FGFR2* demonstrated prognostic significance, and *EGFR* showed the clearest cross‐sample concordance and treatment‐associated relevance. Larger independent validation studies are required before clinical implementation.

## 1 Introduction

Lung cancer (LC) remains one of the most important contributors to global cancer incidence and mortality. According to GLOBOCAN 2022, it was the most frequently diagnosed cancer worldwide, with almost 2.5 million new cases (12.4% of all cancers), and the leading cause of cancer death, accounting for approximately 1.8 million deaths (18.7% of all cancer‐related deaths) [1]. In Türkiye, lung cancer continues to represent a major cancer burden, particularly among men. In 2022, the age‐standardized incidence rate for lung cancer was 68.0 per 100 000 in men and 13.5 per 100 000 in women, while the corresponding age‐standardized mortality rates were 66.3 and 10.1 per 100 000, respectively [2]. LC is broadly classified into two principal categories: small‐cell lung cancer (SCLC) and non‐small‐cell lung cancer (NSCLC). NSCLC constitutes the predominant histological category, accounting for close to 80% of all lung cancers, whereas SCLC represents a smaller but biologically more aggressive subtype characterized by rapid growth and early metastatic dissemination. According to the World Health Organization classification, NSCLC comprises three major histological subtypes—adenocarcinoma, squamous cell carcinoma, and large cell carcinoma. Among these, adenocarcinoma is the most common subtype and accounts for approximately 40% of all lung cancers [3]. Advanced NSCLC is a molecularly heterogeneous disease, and improved biomarker‐based stratification is therefore fundamental to diagnosis, prognostic assessment, and treatment selection. Interpatient diversity, intratumoral heterogeneity, and genomic differences between primary and metastatic lesions may all affect biomarker profiles, treatment response, and prognosis, underscoring the importance of comprehensive molecular testing in contemporary NSCLC management [4, 5]. Accordingly, molecular biomarkers are now integral to modern NSCLC management, serving not only to identify therapeutically actionable alterations, but also to improve prognostic assessment and to inform longitudinal evaluation of treatment response. As NSCLC care continues to shift toward precision oncology, biomarker testing has assumed a pivotal role in individualized therapeutic decision‐making [6, 7]. However, despite remaining the reference standard for histological and molecular assessment, tissue biopsy is limited by procedural invasiveness, restricted repeatability, insufficient material for broad biomarker testing in a subset of patients, and incomplete representation of tumor heterogeneity, thereby constraining its role in longitudinal monitoring [8].

Liquid biopsy has emerged as a minimally invasive platform for the detection of circulating tumor‐derived biomarkers in peripheral blood, including CTCs, cfDNA/ctDNA, cfRNA, microRNA, and extracellular vesicle–associated analytes. By enabling serial sampling, it offers the opportunity to monitor tumor dynamics over time and to capture evolving molecular alterations associated with disease progression and treatment resistance [8, 9]. However, blood‐based liquid biopsy may not fully capture local molecular events arising from the airway epithelium and pulmonary tumor microenvironment. EBC therefore represents a promising non‐invasive, organ‐specific biofluid, obtained by cooling exhaled air and collecting respiratory droplets enriched with volatile and non‐volatile biomolecules, including nucleic acids, proteins, and metabolites, thereby offering complementary insight into lung‐derived molecular alterations [10, 11]. Because EBC directly reflects the respiratory compartment, it may provide organ‐specific molecular information that complements, rather than simply duplicates, plasma‐based liquid biopsy findings. Current evidence indicates that EBC captures localized airway‐derived signals that may be underrepresented in systemic biofluids, thereby offering additional insight into pulmonary and tumor‐microenvironment–related processes. Prior studies have demonstrated the feasibility of EBC‐based biomarker assessment in lung cancer, including the detection of transcriptomic markers such as microRNAs, mRNA isoforms, and other cell‐free nucleic acids, as well as inflammatory and oxidative stress–related mediators. Collectively, these observations support the view that EBC may yield clinically informative molecular data relevant to lung cancer detection, disease characterization, and, in selected contexts, association with clinicopathological features [10, 11, 12].

Lung adenocarcinoma (LUAD) develops through a multistep molecular process characterized by substantial genetic heterogeneity and cumulative genetic and epigenetic alterations, which together dysregulate signal transduction, transcriptional programs, apoptotic balance, and metabolic pathways that support tumor progression and therapeutic resistance [13, 14]. Among the molecular determinants most strongly implicated in NSCLC biology, *EGFR* has a central role in tumor development and therapeutic stratification. Activating *EGFR* alterations drive downstream signaling pathways that promote cell proliferation, survival, and disease progression, while aberrant *EGFR* activation is clinically associated with aggressive tumor behavior, treatment resistance, and the need for mutation‐directed targeted therapy [15].

*FGFR2* dysregulation has been implicated in lung cancer progression and may contribute to aggressive tumor behavior and adverse clinical outcome, supporting its relevance as a potential therapeutic target in NSCLC [16]. Activation of the PI3K pathway through *PIK3CA* alteration has been linked to tumor progression in NSCLC and, particularly in EGFR‐mutant disease, to poorer clinical outcome and shorter survival under EGFR‐TKI therapy [17, 18]. *TP53* alterations are frequent in advanced NSCLC and define a distinct molecular subset characterized by associations with smoking history, higher PD‐L1 expression, increased tumor mutational burden, and specific co‐mutational patterns, supporting their relevance to aggressive tumor biology and clinical stratification [19, 20]. *PTEN* negatively regulates PI3K/AKT signaling and is frequently dysregulated in NSCLC through loss of expression, mutation, or functional impairment, contributing to tumor progression and resistance to therapy [21]. *STK11* alterations contribute to impaired cellular energy regulation and unfavorable therapeutic outcomes [22], whereas *SMAD4* inactivation has been associated with genomic instability and altered treatment response [23].

In this context, the present study was designed to address three distinct but related analytical questions. First, we evaluated the diagnostic relevance of EBC‐derived cfRNA expression by testing whether selected gene‐expression profiles could distinguish patients with advanced LUAD from healthy controls. Second, we examined the prognostic significance of these markers by assessing their association with overall survival. Third, we explored their potential predictive relevance by analyzing treatment‐related outcome differences, particularly in patients receiving chemotherapy‐based regimens. To further clarify the biological and translational value of EBC, we also compared EBC‐derived findings with paired plasma cfRNA data in a subset of participants. Thus, rather than broadly reassessing established NSCLC molecular biology, this study specifically investigates whether EBC‐based cfRNA profiling can provide clinically meaningful diagnostic, prognostic, and treatment‐associated information in advanced LUAD using a minimally invasive respiratory sampling approach.

## 2 Materials and Methods

### 2.1 Study Population

This case–control study enrolled 40 patients with histologically confirmed primary lung adenocarcinoma at advanced clinical stages (Stage IIIB or IV), all of whom had an Eastern Cooperative Oncology Group (ECOG) performance status consistent with eligibility for systemic therapy. Patients were recruited from the Department of Chest Diseases, Faculty of Medicine, Ege University. A control cohort of 20 age‐ and sex‐matched healthy volunteers, with no prior history of malignancy or chronic pulmonary disease, was also included for comparative analysis. Comprehensive demographic, epidemiological, and clinical characteristics—including age, sex, smoking status, disease stage, treatment regimen, and comorbidities—were systematically documented using standardized case report forms. All procedures involving human participants were conducted in accordance with the ethical standards of the Declaration of Helsinki and its later amendments. The study protocol was reviewed and approved by the Ege University Institutional Medical Research Ethics Committee (Approval No. 22‐5.1/29). Written informed consent was obtained from all participants prior to enrolment.

### 2.2 Collection of Clinical Samples (EBC and Plasma)

EBC samples were obtained from all participants, comprising 40 patients with advanced‐stage lung adenocarcinoma and 20 healthy, age‐ and sex‐matched controls. To assess the concordance of cell‐free RNA (cfRNA) expression profiles between EBC and liquid biopsy, peripheral blood samples were additionally collected from a subset of participants, including 10 patients with histologically confirmed lung adenocarcinoma and 10 healthy controls. All clinical specimens were obtained on a single day, during one clinical appointment, and within the same morning timeframe.

#### 2.2.1 EBC Sample Collection

EBC samples (2 mL) were collected using a custom‐designed glass EBC condenser, as previously described by our group [24]. To minimize pre‐analytical variability and potential circadian influences on EBC composition, all samples were collected under standardized conditions in the morning hours, within the same predefined time window, and after an overnight fast, in accordance with previous reports indicating that collection timing and physiological status may affect breath condensate constituents [25, 26]. Participants were instructed to breathe tidally through a mouthpiece connected to the condenser for the designated collection period, while care was taken to avoid salivary contamination. Immediately after collection, condensate samples were transferred into sterile, DNA‐ and RNA‐free 15 mL polypropylene tubes and stored at −20°C until RNA extraction.

#### 2.2.2 Peripheral Blood Sample Collection

Peripheral venous blood (4 mL) was collected into EDTA‐coated tubes from the designated subset of participants. To ensure temporal comparability between specimen types, peripheral blood and EBC samples were collected on the same day, during the same clinical visit, and within the same morning time window under fasting conditions. This standardized sampling strategy was implemented to minimize potential variability related to circadian or short‐term physiological fluctuations and to reduce the likelihood that differences observed between plasma and EBC gene expression profiles were attributable to differences in sampling time rather than biological matrix‐specific features. To minimize cfRNA degradation, all blood samples were processed within 2 h of collection. Plasma was separated by centrifugation at 2000 × *g* for 10 min at 4°C, and the supernatant was subsequently transferred to fresh, sterile centrifuge tubes. A second centrifugation step at 13000 × *g* for 5 min at 4°C was then performed to remove residual cellular debris. The clarified plasma aliquots were stored at −80°C until downstream molecular analyses.

### 2.3 Gene Expression Analysis

Total RNA was extracted from EBC and plasma samples of both patients and healthy controls using the *PureLink RNA Mini Kit* (Thermo Fisher Scientific, USA) according to the manufacturer's protocol. RNA purity and concentration were assessed using a *NanoDrop 1000* spectrophotometer (Thermo Scientific, USA), ensuring that A260/A280 ratios fell within the acceptable range for downstream applications. First‐strand complementary DNA (cDNA) synthesis was performed from purified RNA using the *EvoScript Universal cDNA Master Kit* (Roche Applied Science, Germany), following the manufacturer's guidelines. Quantitative real‐time reverse transcription polymerase chain reaction (RT‐qPCR) was conducted to determine the expression levels of *EGFR*, *FGFR2*, *PIK3CA*, *PTEN*, *SMAD4*, *STK11*, and *TP53*. Amplification reactions were carried out with the *RT SYBR Green qPCR Mastermix Kit* (Roche Applied Science, Germany) using gene‐specific primers (Table 1) on a *LightCycler 480 Instrument II* (Roche Diagnostics, Switzerland). The housekeeping gene *GAPDH* served as the internal reference for normalization of expression data. Relative gene expression changes were calculated using the 2^−ΔΔCT^ method [27]. All assays were performed in triplicate to ensure reproducibility, and appropriate negative controls were included to detect potential contamination.

**TABLE 1:** Gene‐specific primers.

Gene	Primers	Amplicon size (bp)
*EGFR*	F: AACACCCTGGTCTGGAAGTACG	106
	R: TCGTTGGACAGCCTTCAAGACC	
*STK11*	F: CTACTGAGGAGGTTACGGCACA	128
	R: ACGCTGTCCAGCATTTCCTGCA	
*FGFR2*	F: GTGCCGAATGAAGAACACGACC	161
	R: GGCGTGTTGTTATCCTCACCAG	
*PIK3CA*	F: GAAGCACCTGAATAGGCAAGTCG	146
	R: GAGCATCCATGAAATCTGGTCGC	
*PTEN*	F: TGAGTTCCCTCAGCCGTTACCT	138
	R: GAGGTTTCCTCTGGTCCTGGTA	
*SMAD4*	F: CTACCAGCACTGCCAACTTTCC	106
	R: CCTGATGCTATCTGCAACAGTCC	
*TP53*	F: CCTCAGCATCTTATCCGAGTGG	128
	R: TGGATGGTGGTACAGTCAGAGC	
*GAPDH*	F: TCAAGGCTGAGAACGGGAAG	87
	R: CGCCCCACTTGATTTTGGAG	

### 2.4 Statistical Analysis

Quantitative gene expression data were analyzed by normalizing the cycle threshold (CT) values obtained from the *LightCycler 480 Instrument II* to the reference housekeeping gene *GAPDH*. Relative expression levels were calculated using the 2^−ΔΔCT^ method [27], with normalization to the control group. The normalized values were subsequently log₂‐transformed prior to statistical evaluation. For differential expression analyses, comparisons of each target gene between patient and control groups were performed using one‐way analysis of variance (ANOVA), followed by Dunnett's multiple‐comparison test, with the control group as the reference comparator. Differential expressions were defined as an absolute fold change > 2 together with a *p* value < 0.05. Heat maps were generated to visualize fold‐change patterns across study groups. The correlation between cfRNA expression levels in EBC and matched liquid biopsy plasma samples was assessed using simple linear regression analysis, with an *R* ^2^ value > 0.70 considered indicative of a strong and significant association. Receiver operating characteristic (ROC) curve analysis was performed using 2^−ΔΔ*CT*^ values for the seven target genes (*EGFR*, *FGFR2*, *PIK3CA*, *PTEN*, *SMAD4*, *STK11*, *TP53*) to evaluate their diagnostic performance, expressed as area under the curve (AUC), sensitivity, and specificity in distinguishing lung adenocarcinoma patients from healthy controls. Kaplan–Meier survival curves were generated to examine the prognostic relevance of significantly dysregulated genes detected in EBC at diagnosis, with survival differences compared using the log‐rank test. Associations between gene expression profiles and clinicopathological features were also assessed. All statistical analyses were conducted using GraphPad Prism software, version 10.0 (GraphPad Software, USA). A two‐tailed *p* < 0.05 was considered significant.

## 3 Results

### 3.1 Epidemiological and Clinical Characteristics

The study cohort consisted of 40 patients with histopathologically confirmed advanced lung adenocarcinoma and 20 healthy controls without a history of malignancy. The patient group was predominantly male, comprising 29 men (72.5%) and 11 women (27.5%), whereas the control group showed an equal sex distribution. The mean age differed modestly between groups, with a mean age of 64.05 ± 9.90 years in the patient cohort and 59.65 ± 4.65 years in the control group. As summarized in Table 2, the clinical profile of the patient cohort reflected a substantial burden of advanced disease. Only 10.0% of patients were diagnosed at stage IIIB, whereas 47.5% and 42.5% were classified as stage IVA and IVB, respectively. Metastatic dissemination was also pronounced, with 60.0% of patients presenting with multiple‐organ metastases, 20.0% with solitary metastasis, and only 20.0% showing no detectable metastasis. With respect to treatment, 45.0% received chemotherapy alone, 15.0% received combined chemotherapy and immunotherapy, 15.0% were treated with tyrosine kinase inhibitors, and 25.0% did not receive systemic therapy (Table 2). Overall, these findings indicate that the study population represents a biologically heterogeneous and clinically high‐risk cohort of patients with advanced lung adenocarcinoma.

**TABLE 2:** Demographic and clinical characteristics of patients diagnosed with lung adenocarcinoma and the control group.

		Lung adenocarcinoma *n* = 40 (%)	Healty individuals = 20 (%)	*p*
Gender	Female	11 (27.5)	10 (50.0)	0.0962
	Male	29 (72.5)	10 (50.0)	
Age (years)	Female	66.45 ± 11.25	59.86 ± 7.79	0.2438
	Male	63.14 ± 8.94	57.15 ± 12.28	0.1937
Diagnosis	Stage IIIB	4 (10.0)		
	Stage IVA	19 (47.5)		
	Stage IVB	17 (42.5)		
Treatment	CT	18 (45.0)		
	CT IT	6 (15.0)		
	TKI	6 (15.0)		
	Untreated	10 (25.0)		
Metastasis	Multiple	24 (60.0)		
	Solitary	8 (20.0)		
	Not Detected	8 (20.0)		

*Note:* This study included 40 patients diagnosed with lung adenocarcinoma and 20 healthy individuals as controls. In the lung adenocarcinoma group, 72.5% were male with a mean age of 63.14 ± 8.94 years, compared to an equal gender distribution and a younger mean age of 57.15 ± 12.28 years in the healthy control group. The majority of lung adenocarcinoma patients were diagnosed at advanced stages IVA (47.5%) and IVB (42.5%), with 60% presenting multiple organ metastases. Treatment modalities among the patients included chemotherapy (45%), chemotherapy combined with immunotherapy (15%), tyrosine kinase inhibitors (15%), and 25% did not receive any systemic treatment. These demographic and clinical characteristics highlight the aggressive nature of advanced lung adenocarcinoma and emphasize the need for targeted therapies and comprehensive management strategies to improve patient outcomes.

Abbreviations: CT, chemotherapy; IT, immunotherapy; TKI, tyrosine kinase inhibitors.

### 3.2 Gene Expression Findings

**FIGURE 1:**
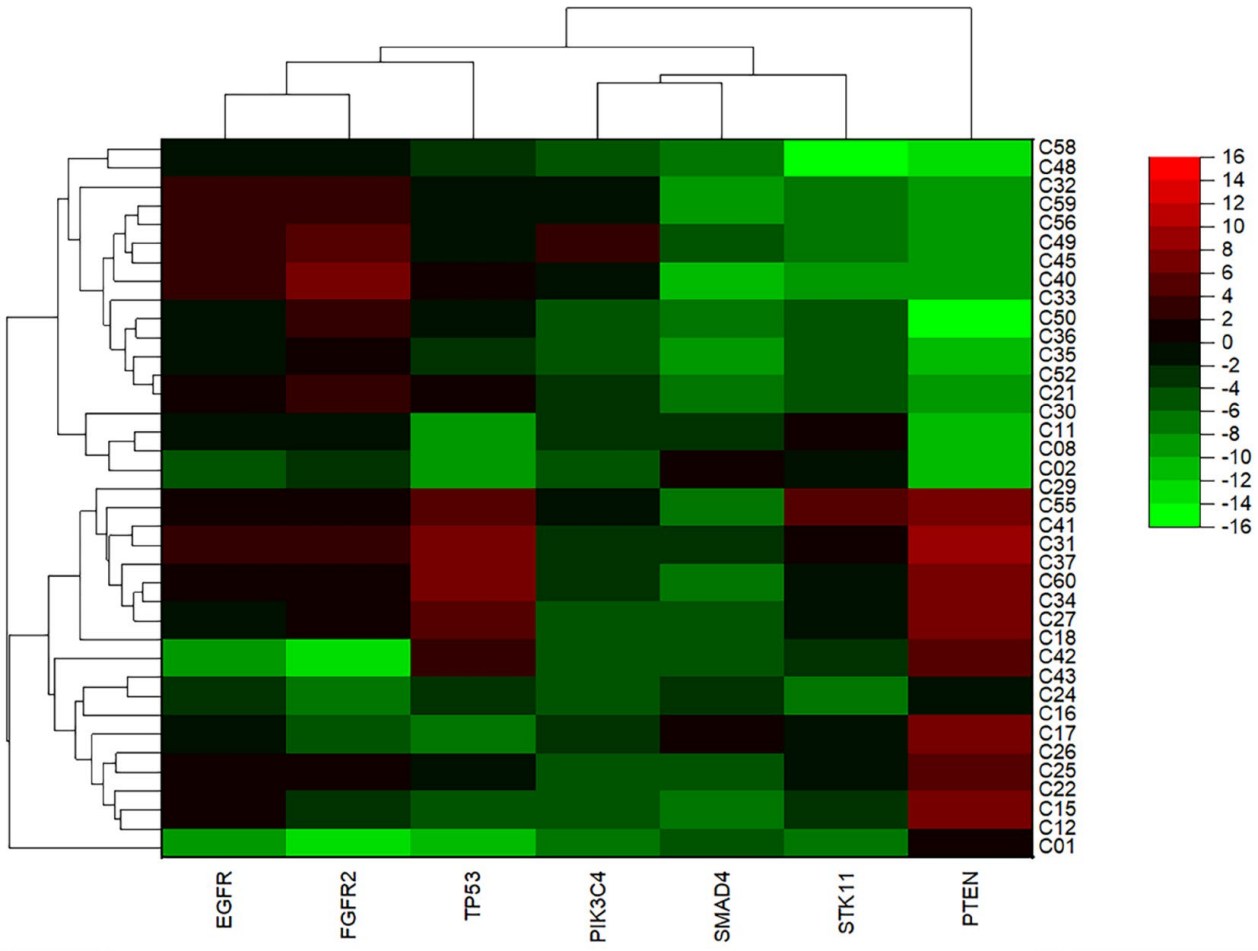
Heat Map of Gene Expression Levels in EBC Samples from NSCLC Patients. The heat map is illustrating the expression levels of seven target genes (*EGFR*, *FGFR2*, *PIK3CA*, *PTEN*, *SMAD4*, *STK11*, and *TP53*) in EBC samples obtained from patients diagnosed with NSCLC. The heat map reveals a distinct pattern of gene expression in NSCLC patients, highlighting significant upregulation of *PTEN*, *TP53*, *SMAD4*, and *FGFR2* compared to healthy controls. These alterations suggest potential roles of these genes in the pathogenesis and progression of NSCLC.

**FIGURE 2:**
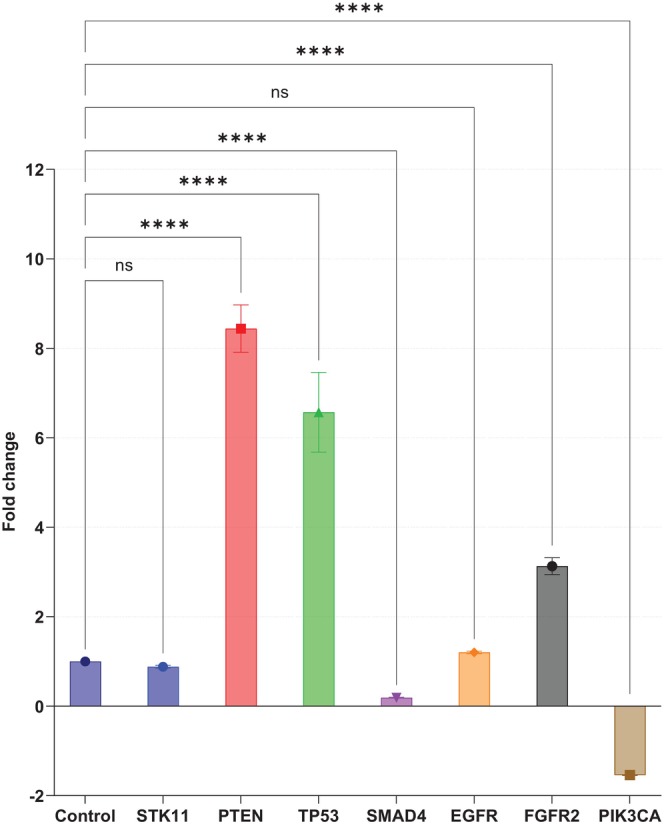
Fold Changes in Gene Expression Levels in EBC Samples Compared to the Control Group. It shows fold changes in gene expression levels of seven target genes (*EGFR, FGFR2, PIK3CA, PTEN, SMAD4, STK11*, and *TP53*) in EBC samples from NSCLC patients compared to healthy control individuals. Significant expression of key oncogenes and tumor suppressor genes in NSCLC provides insight into their potential as diagnostic and prognostic biomarkers.

**FIGURE 3:**
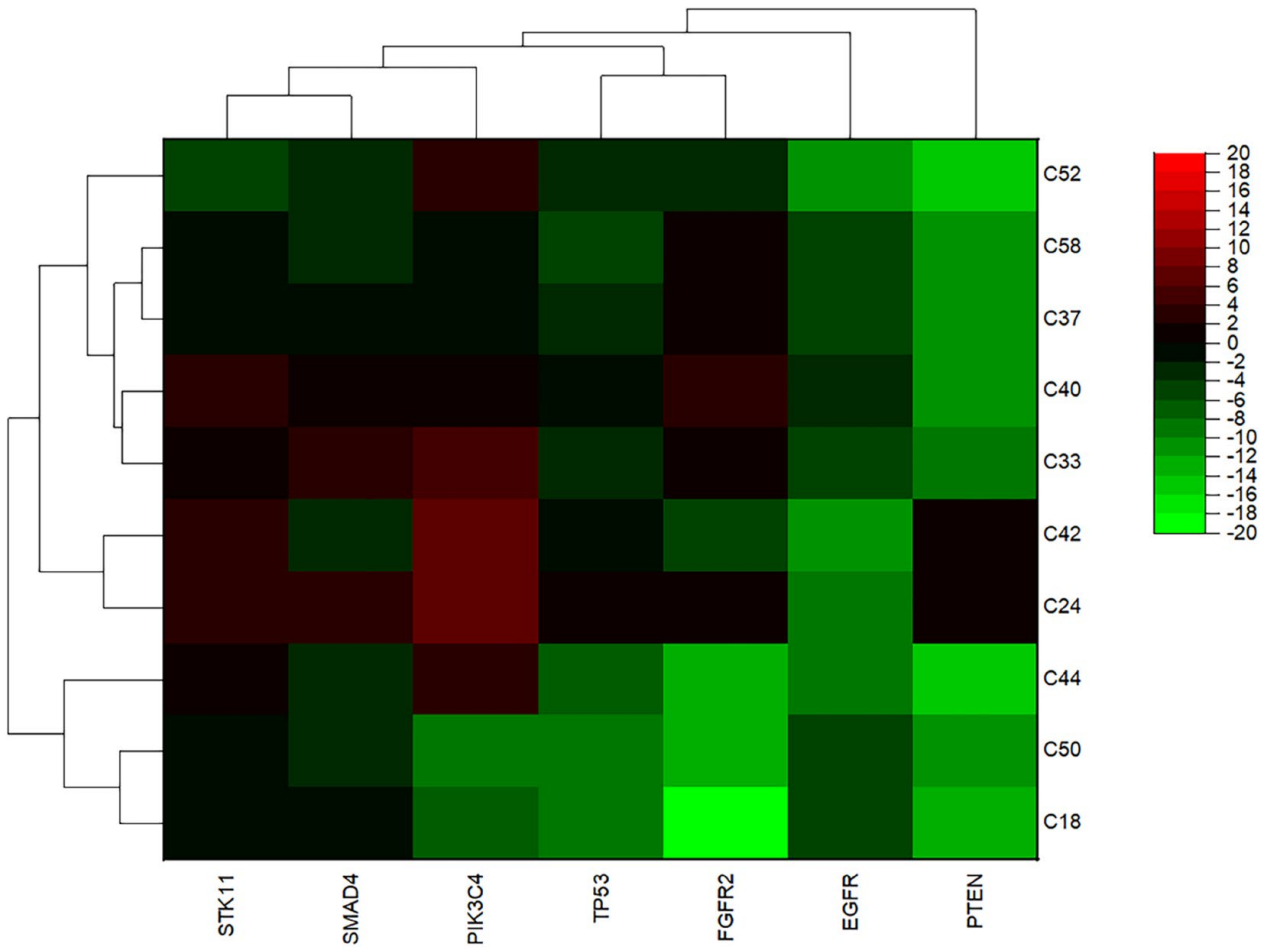
Heat Map of Gene Expression Levels in Plasma Samples from NSCLC Patients. Heat map is illustrating the expression levels of seven target genes (*EGFR*, *FGFR2*, *PIK3CA*, *PTEN*, *SMAD4*, *STK11*, and *TP53*) in plasma samples obtained from patients diagnosed with NSCLC. *PIK3CA* and *EGFR* may play significant roles in NSCLC biology and may serve as valuable biomarkers for disease monitoring and prognosis.

**FIGURE 4:**
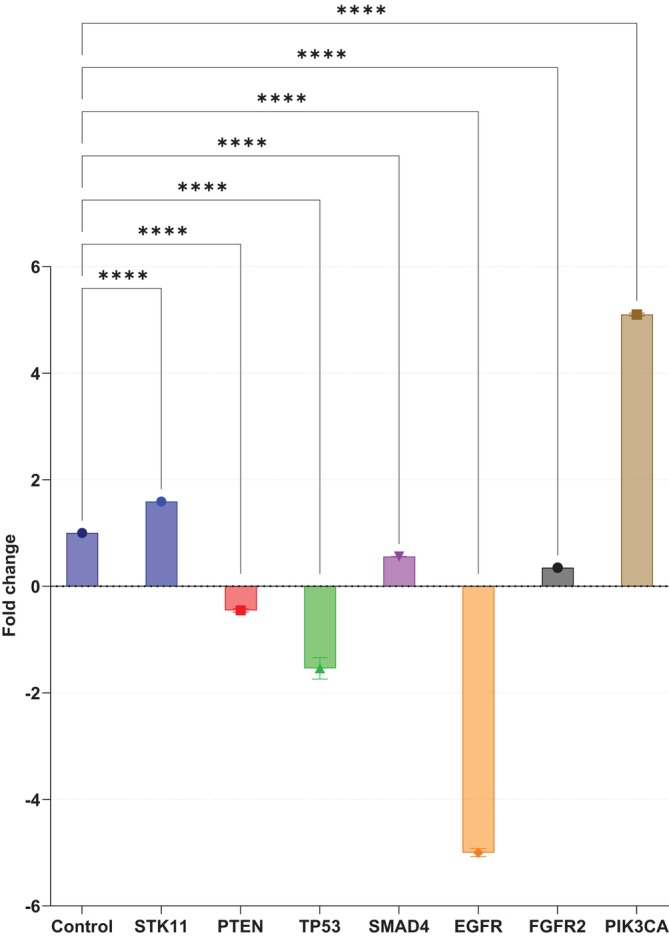
Fold Changes in Gene Expression Levels in Plasma Samples Compared to the Control Group. It shows fold changes in gene expression levels of seven target genes (*EGFR, FGFR2, PIK3CA, PTEN, SMAD4, STK11*, and *TP53*) in plasma samples obtained from patients diagnosed with NSCLC compared to healthy control individuals. There was a significant five‐fold increase in *PIK3CA* expression and a significant five‐fold decrease in *EGFR* expression in NSCLC patients. These findings suggest differential expression of key oncogenes and tumor suppressor genes in the plasma of NSCLC patients, underscoring their potential roles as biomarkers for disease diagnosis and prognosis.

**FIGURE 5:**
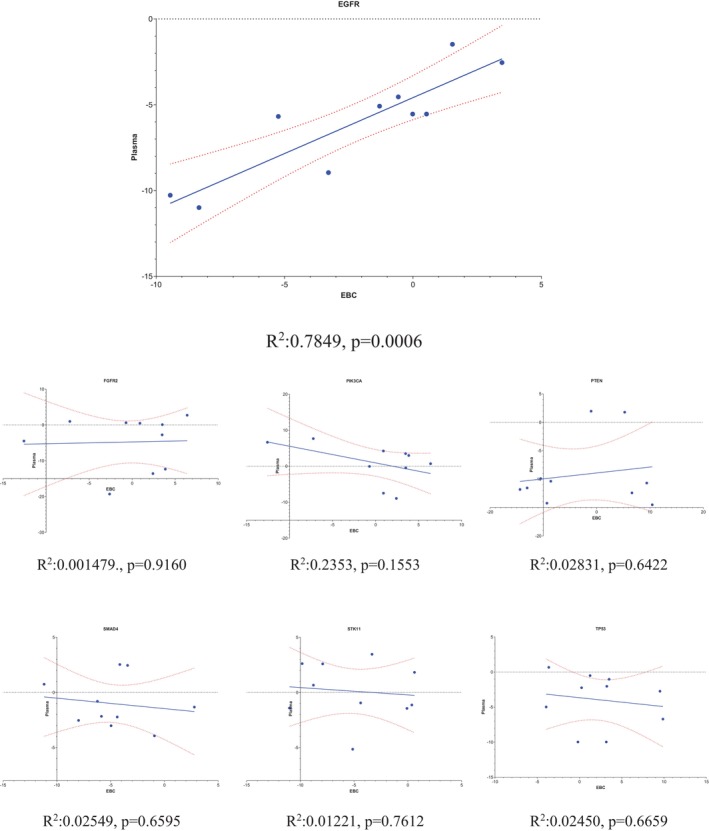
Correlation Graph Between Fold Changes in Gene Expression Levels in EBC and Plasma Samples. The plot demonstrates a strong positive correlation (*R* ^2^ = 0.7849, *p* = 0.0006), indicating that *EGFR* expression levels in EBC closely mirror those in plasma. This significant correlation underscores the potential of *EGFR* as a reliable biomarker accessible through non‐invasive EBC sampling, facilitating its use in both diagnostic and prognostic evaluations of NSCLC.

RNA of sufficient quality for downstream analysis was successfully isolated from all EBC samples and from the plasma subgroup, supporting the feasibility of EBC as a source of cell‐free RNA for molecular profiling in advanced lung adenocarcinoma. Across the seven target genes, the observed expression changes were not uniform; instead, EBC and plasma samples exhibited distinct and selective molecular profiles (Figures 1, 2, 3, 4, 5).

#### 3.2.1 EBC‐Derived Gene Expression

In EBC samples, the most prominent alterations were observed in *PTEN*, *TP53*, and *FGFR2*. Compared with healthy controls, *PTEN* expression showed the highest increase, with an 8.44‐fold elevation, followed by *TP53* with a 6.57‐fold increase and *FGFR2* with a 3.13‐fold increase. In contrast, *PIK3CA* expression decreased by 1.54‐fold. *SMAD4* expression was also significantly altered, whereas *EGFR* and *STK11* did not demonstrate significant differences (Table 3; Figures 1 and 2). These data indicate that EBC captures selected molecular alterations related to tumor suppressor pathways, growth factor signaling, and the PI3K axis in advanced lung adenocarcinoma.

**TABLE 3:** Fold changes of gene expressions in EBC samples compared to the control group.

Gene	Fold change	*p*
*PIK3CA*	−1.54	< 0.0001
*SMAD4*	0.19	0.0082
*STK11*	0.88	0.9969
*EGFR*	1.20	0.9470
*FGFR2*	3.13	< 0.0001
*TP53*	6.57	< 0.0001
*PTEN*	8.44	< 0.0001

*Note:* In exhaled breath condensate samples from NSCLC patients, *PIK3CA* and *SMAD4* genes were downregulated, exhibiting fold changes of −1.54 (*p* < 0.0001) and 0.19 (*p* = 0.0082), respectively. Conversely, *FGFR2*, *TP53*, and *PTEN* genes were markedly upregulated, with fold changes of 3.13, 6.57, and 8.44 (all *p* < 0.0001). No significant changes were observed for *STK11* (fold change = 0.88, *p* = 0.9969) and *EGFR* (fold change = 1.20, *p* = 0.9470). These significant alterations in gene expression highlight the potential of *FGFR2*, *TP53*, and *PTEN* as valuable diagnostic and prognostic biomarkers in advanced lung adenocarcinoma.

#### 3.2.2 Plasma‐Derived Gene Expression

The plasma cfRNA profile differed in part from that observed in EBC. The most notable findings were a marked increase in *PIK3CA* expression (5.10‐fold) and a pronounced reduction in *EGFR* expression (5.00‐fold). Additional significant changes were identified for *TP53*, *PTEN*, *FGFR2*, *SMAD4*, and *STK11*, although the magnitude and direction of these changes differed from the EBC profile (Table 4; Figures 3 and 4). These findings suggest that EBC and plasma provide complementary, rather than interchangeable, information regarding tumor‐associated molecular alterations.

**TABLE 4:** Fold changes in gene expression levels in plasma samples compared to the control group.

Gene	Fold change	*p*
*EGFR*	−5.00	< 0.0001
*TP53*	−1.54	< 0.0001
*PTEN*	−0.45	< 0.0001
*FGFR2*	0.35	< 0.0001
*SMAD4*	0.56	< 0.0001
*STK11*	1.59	< 0.0001
*PIK3CA*	5.10	< 0.0001

*Note:* In plasma samples from NSCLC patients, *EGFR* gene expression was significantly downregulated, exhibiting fold changes of −5.00 (*p* < 0.0001). Conversely, *PIK3CA* gene expression was markedly upregulated, with fold changes of 5.10 (*p* < 0.0001). These significant alterations in gene expression underscore the potential of these genes as diagnostic and prognostic biomarkers in advanced lung adenocarcinoma.

#### 3.2.3 Relationship Between EBC and Plasma Expression Profiles

When expression profiles from EBC and plasma were compared, *EGFR* was the only gene demonstrating a strong and significant positive correlation between the two specimen types (R^2^ = 0.7849, *p* = 0.0006). No comparable correlations were observed for the remaining genes (Figure 5). This pattern suggests that EBC is not merely a passive surrogate of systemic circulation but may instead reflect molecular signals more closely associated with the respiratory tract and local tumor microenvironment.

### 3.3 Diagnostic Performance of Gene Expression Profiles

**FIGURE 6:**
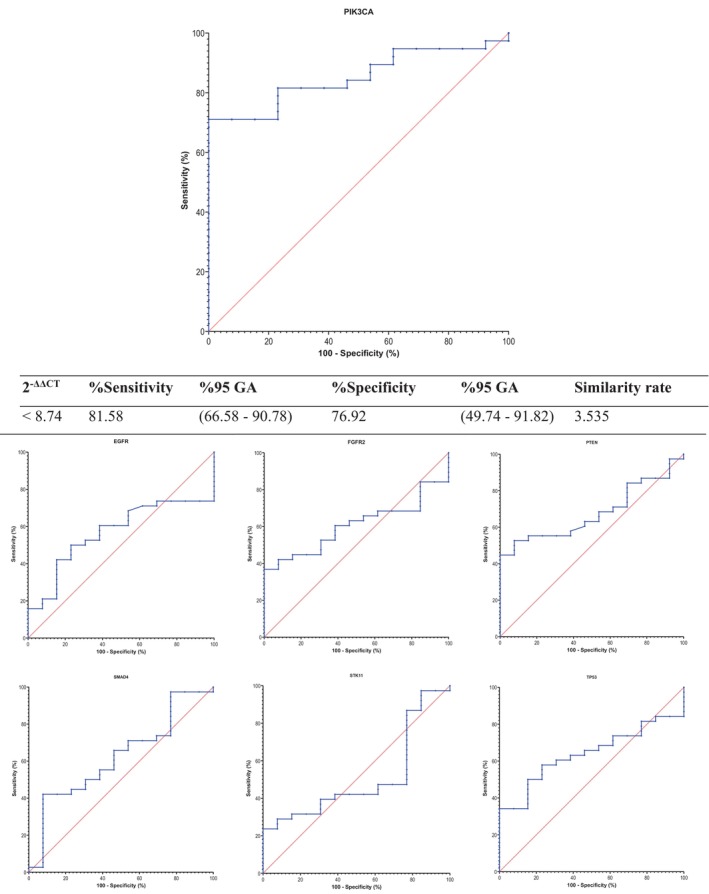
ROC Analysis of NSCLC Patients. It depicts the Receiver Operating Characteristic (ROC) curve analysis for assessing the diagnostic performance of the seven target genes (*EGFR*, *FGFR2*, *PIK3CA*, *PTEN*, *SMAD4*, *STK11*, and *TP53*) in distinguishing NSCLC patients from healthy controls based on their 2^−ΔΔCT^ expression values. The ROC curve for *PIK3CA* is highlighted, demonstrating an Area Under the Curve (AUC) of 0.8522 (*p* = 0.0002), indicating excellent diagnostic accuracy. The sensitivity and specificity for *PIK3CA* at the optimal threshold of 8.74 are 81.58% and 76.92%, respectively. Other genes did not exhibit significant AUC values, suggesting limited diagnostic utility in this cohort. The ROC curve provides a graphical representation of the trade‐off between sensitivity and specificity for each gene, with the *PIK3CA* gene showing the highest potential as a diagnostic biomarker for NSCLC.

The diagnostic performance of the seven target genes was evaluated by ROC curve analysis. Among the genes examined, only *PIK3CA* demonstrated significant discriminatory capacity for distinguishing lung adenocarcinoma patients from healthy controls. At the optimal threshold value of 8.74, *PIK3CA* achieved a sensitivity of 81.58% and a specificity of 76.92%. The area under the curve was 0.8522, indicating strong diagnostic performance (*p* = 0.0002, [Figure 6]). No significant diagnostic utility was identified for the other six genes. These findings highlight *PIK3CA* as the most promising diagnostic candidate biomarker within the analyzed EBC‐based cfRNA panel.

### 3.4 Association Between Gene Expression and Metastatic Status

No significant differences in gene expression were detected between patients with organ metastases and those without metastases. Similarly, when metastatic burden was further stratified as solitary versus multiple‐organ involvement, none of the seven genes showed significant differential expression. These findings suggest that the investigated genes may be more closely associated with the diagnostic and prognostic biology of advanced lung adenocarcinoma than with the presence or extent of metastatic dissemination.

### 3.5 Survival Analysis

**FIGURE 7:**
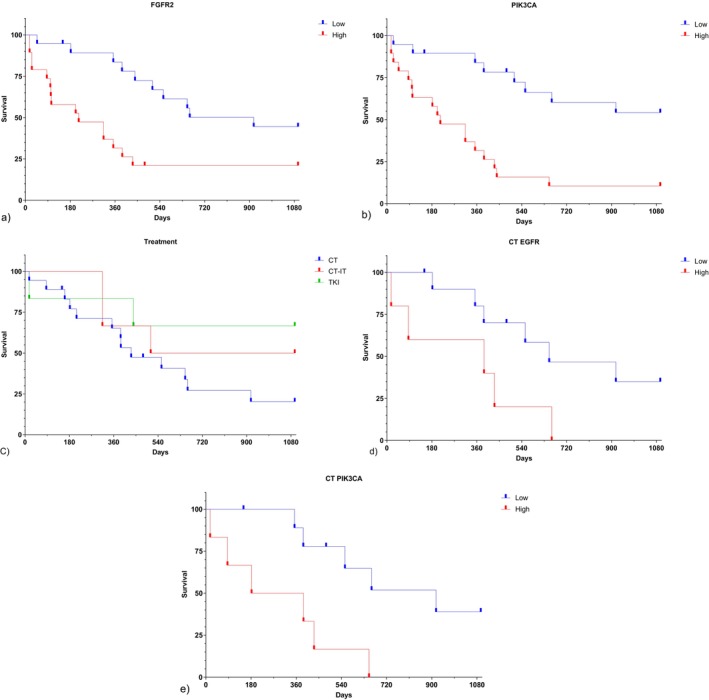
Kaplan–Meier overall survival curves of patients with NSCLC according to gene expression and treatment strata. (a) Overall survival stratified by FGFR2 expression in EBC samples, showing significantly longer survival in the low‐expression group than in the high‐expression group (*p* = 0.007; median survival, 917 vs. 214 days). (b) Overall survival stratified by PIK3CA expression in EBC samples, demonstrating improved survival in patients with low expression compared with those with high expression (*p* < 0.001; median survival, 917 vs. 285 days; 1000‐day survival, 52.5% vs. 10%). (c) Overall survival according to treatment modality, including chemotherapy, chemotherapy plus immunotherapy, tyrosine kinase inhibitor therapy, and no treatment; patients receiving chemotherapy plus immunotherapy showed the most favorable survival pattern. (d) Overall survival among chemotherapy‐treated patients stratified by EGFR expression in EBC samples, with longer survival observed in the low‐expression group (*p* = 0.048; median survival, 650 vs. 389 days). (e) Overall survival among chemotherapy‐treated patients stratified by PIK3CA expression in EBC samples, showing significantly prolonged survival in the low‐expression group compared with the high‐expression group (*p* = 0.005; median survival, 917 vs. 285 days).

At the end of follow‐up, 13 of the 40 patients (32.5%) were alive, whereas 27 (67.5%) had died. Treatment patterns and individual treatment responses are summarized in Table 5. Kaplan–Meier survival analysis identified *FGFR2* and *PIK3CA* as the only genes significantly associated with overall survival. Patients with low *FGFR2* expressions had a median survival of 917 days, compared with 214 days for those with high *FGFR2* expressions (*p* = 0.007, [Figure 7]). Likewise, low *PIK3CA* expression was associated with a more favorable outcome, with a 1000‐day survival rate of 52.5% in the low‐expression group versus 10.0% in the high‐expression group (*p* < 0.001, [Figure 7]). No significant associations with overall survival were observed for *PTEN*, *TP53*, *SMAD4*, *EGFR*, or *STK11*. These results support the prognostic relevance of *FGFR2* and *PIK3CA* expression in advanced lung adenocarcinoma.

**TABLE 5:** Treatments received and treatment responses of patients diagnosed with lung adenocarcinoma.

Patient	First‐line systemic therapy	Treatment response	Prognosis	Survival
P1	CT	Partial response	Good	Alive
P2	CT	Partial response	Good	Ex
P3	CT	Partial response	Good	Ex
P4	CT	Partial response	Good	Ex
P5	CT	Progressive	Good	Alive
P6	CT	Stable	Good	Alive
P7	CT	Stable	Good	Ex
P8	CT	Stable	Good	Ex
P9	CT	Stable	Good	Ex
P10	CT	Stable	Good	Ex
P11	CT	Stable	Poor	Ex
P12	CT	Stable	Good	Alive
P13	CT	Stable	Good	Ex
P14	CT	Stable	Good	Ex
P15	CT	Stable	Good	Ex
P16	CT	Stable	Poor	Ex
P17	CT	Complete response	Good	Alive
P18	CT	Complete response	Poor	Ex
P19	CT IT	Partial response	Good	Alive
P20	CT IT	Partial response	Good	Alive
P21	CT IT	Partial response	Good	Alive
P22	CT IT	Partial response	Good	Ex
P23	CT IT	Progressive	Good	Ex
P24	CT IT	Progressive	Good	Ex
P25	TKI	Partial response	Good	Ex
P26	TKI	Partial response	Poor	Alive
P27	TKI	Progressive	Good	Alive
P28	TKI	Complete response	Good	Alive
P29	TKI	Complete response	Good	Alive
P30	TKI	Complete response	Poor	Ex
P31	NRT	NA	Good	Alive
P32	NRT	NA	Good	Ex
P33	NRT	NA	Good	Ex
P34	NRT	NA	Good	Ex
P35	NRT	NA	Poor	Ex
P36	NRT	NA	Poor	Ex
P37	NRT	NA	Poor	Ex
P38	NRT	NA	Poor	Ex
P39	NRT	NA	Poor	Ex
P40	NRT	NA	Poor	Ex

*Note:* In this study, among the 40 patients diagnosed with lung adenocarcinoma, 18 received chemotherapy (CT), 6 received a combination of chemotherapy and immunotherapy (CT IT), 6 were treated with tyrosine kinase inhibitors (TKI), and 10 did not receive any systemic treatment (NRT). Treatment responses varied, with partial responses observed in 9 patients, complete responses in 5 patients, stable disease in 14 patients, and progressive disease in 5 patients, particularly among those receiving CT alone or no treatment.

Abbreviations: CT, chemotherapy; IT, immunotherapy; NRT, not receive treatment; TKI, tyrosine kinase inhibitor.

### 3.6 Treatment Response and Gene Expression

When survival was analyzed according to treatment modality, patients receiving combined chemotherapy and immunotherapy exhibited longer overall survival than those treated with chemotherapy alone, tyrosine kinase inhibitors, or no systemic therapy (Figure 7; Table 5). Within the subgroup treated with chemotherapy alone, gene‐expression‐dependent survival differences became more evident. Patients with low *EGFR* expression had a median survival of 650 days, compared with 389 days in those with high *EGFR* expression (*p* = 0.048, [Figure 7]). Similarly, among chemotherapy‐treated patients, low *PIK3CA* expression was associated with a median survival of 917 days, whereas the corresponding value for the high‐expression group was 285 days (*p* = 0.005, [Figure 7]). No significant survival differences were observed according to *STK11*, *PTEN*, *TP53*, *SMAD4*, or *FGFR2* expression levels in the chemotherapy subgroup. Taken together, these findings suggest that *EGFR* and *PIK3CA* expression levels may have value in predicting treatment response and clinical course in patients with advanced lung adenocarcinoma receiving chemotherapy.

## 4 Discussion

The present study shows that EBC‐based cfRNA profiling can capture measurable and clinically relevant transcriptional alterations in advanced NSCLC, while also revealing that different genes contribute to different analytical domains. Among the seven genes evaluated, *PIK3CA* showed the clearest diagnostic signal, *FGFR2* demonstrated prognostic relevance, and *EGFR* emerged as the marker with the most consistent cross‐matrix and treatment‐associated implications. This gene‐specific pattern is important because it suggests that EBC‐derived cfRNA should not be interpreted as a uniform biomarker source, but rather as a platform capable of yielding distinct layers of clinically relevant information depending on the target analyzed. In our cohort, *PTEN* expression was markedly elevated in EBC, with an approximately eightfold increase relative to controls. Although *PTEN* loss is more commonly associated with NSCLC progression and adverse outcome [28, 29, 30], the biological significance of *PTEN* overexpression is less clear. Evidence from endometrial carcinoma suggests that *PTEN* overexpression may promote epithelial–mesenchymal transition through the β‐catenin/Slug axis [31]. Accordingly, while our data do not allow mechanistic inference, the elevated *PTEN* levels observed in a cohort characterized by a high metastatic burden may reflect a more complex role for *PTEN*‐associated signaling in advanced disease biology rather than a simple tumor‐suppressive effect. Similarly, *TP53* expression was substantially increased in EBC, consistent with reports linking missense *TP53* mutations to increased mutant p53 protein accumulation [32]. *FGFR2* expression was also elevated and, importantly, its higher expression was associated with shorter overall survival. This finding accords with prior reports in other tumor types, including metastatic gastric cancer, where FGFR2 dysregulation has been linked to clinical aggressiveness, treatment non‐responsiveness, and poor overall survival [33, 34, 35, 36, 37]. Within our dataset, however, *FGFR2* is notable not merely because it was differentially expressed, but because it contributed to prognostic stratification, thereby distinguishing itself from several other altered genes that lacked comparable survival associations.

One of the most clinically relevant findings of the study was the diagnostic performance of *PIK3CA* in EBC. *PIK3CA* expression showed significant discriminatory capacity for differentiating advanced LUAD cases from healthy controls, with an AUC of 0.8522, sensitivity of 81.58%, and specificity of 76.92%. This result positions *PIK3CA* as the strongest diagnostic candidate within the present EBC‐based panel and suggests that EBC‐derived cfRNA may contain sufficiently robust signals to support minimally invasive molecular discrimination in advanced disease. In addition, low *PIK3CA* expression was associated with prolonged survival, further indicating that this marker may have dual diagnostic and prognostic relevance. This convergence of diagnostic and outcome‐associated information makes *PIK3CA* particularly notable among the genes evaluated in this study. Regarding cross‐matrix comparison, the EBC and plasma expression patterns were not fully concordant. This observation should be interpreted cautiously, because the plasma cfRNA analysis was performed in a relatively small subgroup and was not intended to support broad conclusions regarding overall EBC–plasma equivalence. Therefore, any interpretation of EBC–plasma concordance should remain exploratory, and overgeneralization should be avoided. At the same time, EBC and plasma should be regarded as biologically distinct yet complementary liquid biopsy compartments, as EBC primarily captures organ‐specific, airway‐derived molecular signals from the respiratory tract, whereas plasma more broadly reflects systemic circulating biomarkers; accordingly, the two matrices may be expected to show only partial overlap rather than complete concordance [10]. Despite this limitation, *EGFR* expression showed a strong positive correlation between EBC and plasma (R^2^ = 0.7849, *p* = 0.0006). Rather than implying interchangeability between the two matrices, this finding should be viewed as a preliminary indication that selected markers may be reproducibly measurable across compartments.

The clinical relevance of *EGFR* was also supported by treatment‐stratified survival analysis. In patients treated with chemotherapy alone, low *EGFR* expression was associated with longer survival, in line with prior evidence linking higher *EGFR* activity to treatment resistance and unfavorable outcomes in NSCLC [15, 38]. Low *PIK3CA* expression similarly identified chemotherapy‐treated patients with better survival. These observations suggest that, within the treatment context, EBC‐derived *EGFR* and *PIK3CA* measurements may have predictive value, although this interpretation remains hypothesis‐generating and requires prospective validation. In contrast, *PTEN*, *TP53*, *SMAD4*, and *STK11* did not show significant associations with overall survival in the full cohort, indicating that differential expression alone should not be equated with clinical utility unless supported by outcome‐linked analyses. To reduce conceptual overlap with the introduction, the implications of our findings are best framed here in practical rather than general terms. Our data support the view that EBC‐based cfRNA profiling is unlikely to replace tissue biopsy or validated plasma‐based assays in advanced NSCLC; instead, its most realistic clinical role is as a complementary respiratory liquid biopsy approach. In this setting, EBC may be particularly useful when tissue is limited, when invasive sampling is risky or delayed, or when serial tissue biopsy is impractical. Used alongside plasma liquid biopsy, imaging, and standard pathology, EBC may provide an additional minimally invasive layer of molecular information that can contribute to baseline risk assessment and longitudinal monitoring. This may be especially relevant in advanced‐stage disease, where repeated sampling is often clinically desirable but procedurally challenging [39, 40, 41, 42]. At the same time, the present results should be interpreted within a translational framework rather than as immediately practice‐changing evidence. Before EBC‐based cfRNA profiling can be incorporated into routine precision oncology workflows, multicenter validation studies, standardized pre‐analytical procedures for EBC collection and processing, and independent confirmation of clinically relevant thresholds will be necessary to establish reproducibility and clinical utility [10, 11, 43]. Thus, the principal contribution of this study is not to propose EBC as a replacement technology, but to provide evidence that EBC‐derived cfRNA may serve as a feasible adjunctive biomarker platform in advanced LUAD, with *PIK3CA*, *FGFR2*, and *EGFR* emerging as the most clinically informative signals in the present dataset.

This study has several limitations that should be acknowledged. First, the sample size was relatively modest, particularly for subgroup analyses, which may limit statistical power and the generalizability of the findings. Second, the study was conducted at a single center, and therefore the observed gene‐expression patterns may partly reflect cohort‐specific clinical or pre‐analytical characteristics; multicenter validation will be necessary to confirm reproducibility across broader patient populations. Third, although the diagnostic and prognostic signals identified for selected markers were encouraging, the biomarker thresholds used in the present analyses should be regarded as exploratory and hypothesis‐generating rather than definitive for clinical decision‐making. Independent validation in larger cohorts will be required before clinically actionable cutoffs can be established. In addition, the plasma cfRNA comparison was performed in a relatively small subgroup, and thus cross‐matrix concordance between EBC and plasma should be interpreted cautiously. Another important limitation is that the study focused on cfRNA‐based gene expression and did not include protein‐level validation. We agree that changes at the mRNA level do not necessarily translate directly into corresponding protein abundance or functional activity. Although protein recovery from EBC is technically feasible, its low protein content and susceptibility to pre‐analytical and analytical variability require further optimization and larger sample sets for reliable measurement. Therefore, validation of the present transcript‐level findings at the protein level was beyond the scope of this study and should be addressed in future investigations.

In conclusion, *FGFR2*, *EGFR*, and *PIK3CA* expression levels in EBC appear to carry diagnostic, prognostic, and treatment‐associated information in advanced NSCLC; however, their current value lies in complementing, rather than replacing, established tissue‐ and plasma‐based molecular workflows. Integrated with conventional pathology, plasma liquid biopsy, and imaging, EBC‐based cfRNA profiling may contribute to molecular surveillance and risk stratification in selected patients, but larger independent cohorts and harmonized analytical standards are required before adoption into routine precision oncology practice.

## Author Contributions

**Sabina Latifova:** writing – original draft, formal analysis. **Uygar Genis:** formal analysis. **Haydar Soydaner Karakus:** resources. **Korcan Korba:** data curation. **Su Ozgur:** data curation. **Ceyda Aldag:** data curation. **Tuncay Goksel:** resources. **Levent Pelit:** data curation. **Cumhur Gunduz:** conceptualization, investigation, funding acquisition, writing – review and editing, project administration, supervision. **Asli Tetik Vardarli:** supervision, project administration, writing – review and editing, funding acquisition, investigation, conceptualization.

## Funding

The research project received support from the Ege University Office of Scientific Research Projects (TS‐GAP‐2023‐23878).

## Conflicts of Interest

The authors declare no conflicts of interest.

## Data Availability

The datasets generated and analyzed during the current study are not publicly available due to ethical restrictions and patient confidentiality agreements but are available from the corresponding author on reasonable request. Any data shared will be de‐identified to ensure compliance with institutional ethical guidelines and applicable data protection regulations.
